# Can ageing be slowed? Hormetic and redox perspectives

**Published:** 2011-11-24

**Authors:** L Gaman, I Stoian, V Atanasiu

**Affiliations:** * University of Medicine and Pharmacy, Biochemistry Department, Bucharest, Romania; ** R&D Irist Labmed, 24 Miraslau, Bucharest, Romania

**Keywords:** senescence, anti–aging, antioxidants, hormesis, hormetins

## Abstract

Redox metabolism has long been considered to play important roles in aging and the development of age-related diseases. Both dietary and pharmacological manipulations of redox metabolism have been associated with the extension of lifespan. Increasing new evidence s also suggests that the process of aging may derive from imperfect clearance of oxidatively damaged material. The accumulation of this molecular “garbage”, relatively indigestible, further hinders cellular functions, induces progressive failure of maintenance and repair and increases the probability of death. One important trend in anti–aging strategy is, therefore, to prevent or even revert the accumulation of these oxidatively altered molecules by stimulating the maintenance and repair systems through hormesis. A promising approach for slowing down ageing and achieving a healthy senescence is represented by repeated exposure to various mild stresses (caloric restriction, moderate exercise, nutritional or pharmacological hormetins). This article reviews the potential therapeutic tools available to date for increasing longevity and obtaining and successful ageing from the redox and hormetic perspective.

## Introduction

One of the most popular hypothesis of ageing is represented by the so called ”garbage catastrophe” or the accumulation of damaged molecules, also including the products oxidatively induced, such as oxidised proteins, DNA adducts, lipid peroxides [**1,2**]. Therefore, one important trend in anti–aging strategy is to prevent or even revert the accumulation of oxidatively altered molecules by modulating the cellular redox state.

Various redox–dependent gerontomodulatory approaches include: 1. dietary or pharmacological antioxidant therapy; 2. hormesis based interventions (including caloric restriction, exercise, etc.). These two categories are not completely distinct, overlapping each other at a certain degree (i.e. antioxidants are also included among hormetic agents).

A literature search was conducted by using PubMed and Highwire to collect data from studies on anti–aging therapy. All the data collected was published in English and available up to August 2011. Ageing, oxidative stress, antioxidants, hormesis, hormetins were used as search terms. A supplemental manual search of the references in the identified articles was performed.

### 1. Antioxidant therapy

Even if exogenous antioxidants can reduce the accumulation of oxidized molecules, it is not yet clear if they manage to extend life span [**3**]. Present data are contradictory; scientists are not able to decide if antioxidant anti–ageing therapy is magic or reality. Even if this dilemma exists, at least one thing is obvious: antioxidant supplementation seems unlikely to increase longevity when begun in middle age, but it might be more effective when started in early life [**4,5**].

#### Dietary antioxidants

The evidence for diet linked longevity is substantial. The example of long–lived populations from Okinawa island and rural regions of Mediterranean countries, i.e. Greece, Italy, and Spain, whose diets are mainly based on fruit, vegetables (i.e. orange–yellow root vegetables, green leafy vegetable), pulses, virgin olive oil, fish, and traditional spices rich in natural antioxidants, strongly suggests the therapeutic anti–aging potential of dietary antioxidants [**6,7**]. Other features such as the low levels of saturated fat, low glycemic load, and low caloric intake are also likely to contribute to the anti–ageing benefits of these diets [**8,9**].

Accumulating evidence from animal and human studies indicates that dietary antioxidants might play an important role in increasing life span.

Nectarines, resveratrol and *Aloe vera* extract supplementation of diet extended adult longevity in *Drosophila melanogaster* by decreasing the expression of oxidative stress–response genes, reducing the oxidative damage, or increasing the antioxidant enzymes activity [**10,11**]. An oregano–cranberry mixture also promoted longevity in the Mexican Fruit fly (*Anastrepha ludens*) [**12**].

Few human studies support the same hypothesis that dietary antioxidant supplementation might be beneficial for the slowing of the aging process. Vitamin E may extend the life expectancy of men with 2 years, if they have a dietary vitamin C intake above the median and smoke less than a pack of cigarettes per day [**13**]. A higher intake of green and yellow vegetables was significantly associated with a decreased skin aging measured as extent of facial wrinkles in the crow's–foot area [**14**].

Even if dietary antioxidants have a prolonged effect or not, they might have at least a beneficial influence on the redox status of the organism. For instance, dietary supplementation, for 15 weeks, with 5% and 20% (w/w) of nutritional doses of vitamins C and E, zinc, selenium, and beta–carotenes restored redox homeostasis and improved leukocyte functions, especially in animals with premature aging, by decreasing malondialdehyde and 8–oxo,7,8–dihydro–2'–deoxyguanosine levels in peritoneal leukocytes [**15**]. In some cases, experiments in vertebrates (i.e. rodents) showed no effect of vitamin E on longevity or even shortened the animal life span [**16**].

Among all antioxidants, the non–enzymatic lipophilic ones may play a pivotal role in aging process, especially due to their general good absorption and slow excretion within our body [**17**].

#### Pharmacological antioxidants

Several pharmacological antioxidants have been tested as anti–aging agents in different animal models.

*Nitrone–based free radical trapping agents.* α–phenil–tert–butyl nitrone (PBN) and other nitrone–based free radical trapping agents prolong both the average and maximum life span in mice [**18,19**]. PBN rapidly penetrates to most tissues and suppresses production of ROS by mitochondria and also inhibits gene transduction caused by oxidative insults [**20,21**].

*SOD/catalase mimetic drugs.* Synthetic salen–manganese complexes termed EUK (Eukarion) were developed to mimic activities of the endogenous superoxide dismutase and catalase involved in neutralization of superoxide and hydrogen peroxide, respectively [**22,23**]. Treatment of MnSOD knock–out mice with EUK–8 (mimetic of both antioxidant enzymes) extends the animal lifespan by threefold and rescues the spongiform encephalopathy (Melov–x). Tempol, a stable nitroxide free radical, is another superoxide dismutase mimetic [**24**]. Tempol. significantly increased the life span of ATM–deficient mice (found in a continuous state of oxidative stress) when the diet was given from weaning, but no effect was found when the treatment was started from fertilization [**25**].

*N–acetyl cysteine* (NAC) is another pharmacological thiol–containing antioxidant which acts according to several mechanisms: it detoxifies ROS and can enhance glutathione synthesis [**26**]. Used for 40 years in clinical practice, NAC has been found to be a safe drug, even when used at high doses and long term [**23**]. NAC have been tested as a chemopreventive antioxidant in ATM–deficient mice characterized by a continuous oxidative stress and a high incidence of lymphoid malignancies. NAC significantly increased the life span and reduced both the incidence and multiplicity of lymphoma [**27**].

The results of redox involved gene manipulation are controversial. Ubiquitous overexpression of SOD does not extend life span in rodents [**28**], but does increase it in Drosophila [**29**]. 

However, the influence of antioxidative dietary or pharmacological factors on redox status and their potential in age–related disease prevention has yet to be elucidated in greater detail. 

### 2. Hormesis based interventions

The adaptive response, induced by low doses of otherwise noxious agents, which leads to the improvement of functional capacity of cells and organisms, is known as hormesis [**30,31**]. The modest overcompensation that comes after the disruption of homeostasis in this mild harmful conditions is the key for understanding hormetic mechanisms [**30**]. In toxicology, hormesis is defined as a dose–response reaction characterised by either a J–shaped or an inverted U–shaped dose–response [**30,32**].

Hormesis based interventions represent a recent approach which use the repeated exposure to a mild stress as a way to stimulate the maintenance and repair systems. Many recent findings suggest that healthy aging may be achieved by hormesis through mild and periodic, but not severe or chronic oxidative challenges [**31**].

This new theory suggests that hormesis induced by mild oxidative challenges might be correlated especially with the role of ROS as essential signaling molecules for promoting metabolic health and longevity [**33**].

Stresses that have been reported to improve lifespan or prevent age–related diseases are: caloric restriction [**34**], intermittent fasting [**35,36**], pro–oxidants [**37**], heavy metals [**38**], heat shock [**39**], irradiation (UV–, gamma–, and X–rays) [**40,41**], exercise [**42**], psychosocial stress [**43**].

#### Caloric restriction

Reduction of caloric intake without malnutrition is one of the most consistent anti–aging experimental interventions. Studies on both non-human [**44,45**] and human primates [**46**] showed that *caloric restriction* extands lifespan. Scientists found that 2 biomarkers of longevity (fasting insulin level and body temperature) and DNA damage were decreased by prolonged calorie restriction (6 months) in humans, although protein carbonyl concentrations were not change from baseline [**46**]. Other various mechanisms are invoked by researchers to explain the antiagieng effect of caloric restriction: reduced lipid peroxidation, increased efficiency of oxidative damage repair, enhanced antioxidant defence, decreased mitochondrial free radical generation rate [**47,48,49,50**].

Despite these evidences, caloric restriction is considered by certain critics a questionable approach for the expansion of the lifespan in humans due to certain pitfalls (i.e. decreased quality of life) [**51**], potential negative effects (i.e. hypotension, infertility, osteoporosis, depression and irritability), and thus, low feasibility for most humans [**52**]. Although, a recent calorie restriction study showed that dietary adherence to long–term controlled feeding in overweight men and women is good when all foods are provided and when participants are highly motivated [**53**].

#### Xenohormesis and hormetins

As a response to the critics of caloric restriction, new types of interventions mimicking the effect of caloric restriction or the effect of other stressor while avoiding their negative aspects have been developed. These interventions use a special class of hormetic anti–aging agents known as *chemical mimetics of real stresses*: chemical mimetics of caloric restriction (i.e. 2–deoxy–D–glucose, resveratrol) [**54,55**], chemical mimetics of heat shock (i.e. curcumin) [**56**]. Thus, a new concept has been introduced by Lamming: xenohormesis, which means hormesis induced by biochemical compounds [**57**]. Xenohormetic compounds decrease risk of common age–related conditions, such as cancer, cardiovascular disease, type 2 diabetes, and neurological diseases, so lengthening lifespan [**58**]. These mild stress–inducing molecules are also called hormetins, and they may be nutraceutical or pharmacological.

*Nutraceutical hormetins*. Hormetic mechanisms of ageing may underlie many of the health benefits of a high intake of fruits and vegetables. Several chemicals in foodstuffs, such as vitamins, antioxidants, polyphenols, macro– and microminerals such as iron, iodine, flurine, selenium, copper, zinc, ethanol, etc. have been shown to induce a typical hormetic dose– response [**59,60**]. The beneficial effects of these so called ”hormetins” are mainly achieved through stress induced regulation of various maintenance and repair pathways, including antioxidant protection pathways [**61**]. Exposure to low concentrations of various phytochemicals (resveratrol, tannic acid, juglone) induces potent life–prolonging properties in the nematode worm *Caenorhabditis elegans*, at least in part due to increased resistance to oxidative stress [**62,63**].

Despite experimental advances in hormesis and ageing research, findings in humans are still quite limited.

*Pharmacological hormetins*. There is now strong interest in discovering and developing pharmacological hormetins capable of inducing an increase in lifespan.

Carnitines (carnitine/acetylcarnitine) have recently been demonstrated to be neuroprotective through the activation of hormetic pathways: they modulates redox–dependent mechanisms leading to up–regulation of so called vitagens, genes involved in longevity assurance processes [**64,65,66**]. There is also one study where scientists found that a chronic dose of acetylcarnitine decreased mortality in animals, showing a prolongevity potential [**67**]. Ozone, another candidate for the pharmacological anti–aging hormetins, when used at appropriate doses, promotes the generation of reactive oxygen species and a consequent antioxidant response capable of reversing the oxidative stress [**37**]. Findings of an increased level of antioxidant enzymes during ozone therapy show the hormetic potential of this pharmacological agent.

#### Exercise

Exercise has been also proposed to modulate free radicals as well as the aging process in a way that can be described by the hormesis curve [**68**]. The two end points of the hormesis curve, which are physical inactivity and overtraining, result in increase of both oxidative stress and incidence of age–related disorders [**38,69,70**], while the regular moderate exercise induces a ROS mediated adaptation which results in enhanced activity of antioxidant and damage repair enzymes, lower levels of oxidative damage and prevent various age-associated loss (e.g. decreased skeletal muscle function, cognition) [**38,71,72**].

## Concluding remarks

Janus’s face of free radicals accounts for the dual effect of oxidative stress on ageing: severe oxidative stress accelerates ageing, while mild oxidative stress (low levels of repeted oxidants exposure) improves lifespan. Manipulation of endogenous cellular defense mechanisms such as the antioxidant response, through nutritional or pharmacological antioxidants or hormetins, represents an innovative approach to anti–aging therapeutic intervention.

As a conclusion, do the recent findings suggest that we can have a long and ”succesfull” life or a healthy ageing? 1) Restrict calories, but maintain the nutritional requirements; 2) Eat food items rich in hormetins (i.e. fruits and vegetables); 3) Use supplements containing hormetins (i.e. resveratrol); 4) Have a regular moderate physical activity, but not performing strenous exercise; 5) Avoid exposure to toxic levels of noxious compounds (i.e. heavy metals).

Although none of the antioxidants or hormetins has yet provided clear–cut evidence toward longevity, they have certainly demonstrated certain preventive potential benefits against various age–related diseases. Further studies are required in order to elucidate in greater details the anti–aging efficacy of the redox and hormetic agents.

*Disclosure statement*. There is no conflict of interests and this study has not been supported by any grant.
